# CRISPR-Induced Loss of Connexin 43 Expression Sensitizes KRAS Mutant Cells to Cisplatin

**DOI:** 10.17912/micropub.biology.000681

**Published:** 2022-11-13

**Authors:** Stephen Martin, David Mu

**Affiliations:** 1 Eastern Virginia Medical School

## Abstract

Gap Junction intercellular communication (GJIC) is often dysregulated in cancers, and this dysregulation has been shown to have pro-tumorigenic effects. Connexins (Cxs) are transmembrane proteins that make up gap junctions. Previous studies have indicated that RNA interference (RNAi)-based suppression of Cx43 increases cellular resistance to the chemotherapeutic agent cisplatin. Interestingly, we found that the loss of Cx43 expression induced by the CRISPR-Cas9 technology sensitizes cells to cisplatin in a KRAS mutant-dependent manner.

**Figure 1. CRISPR-induced Cx43 mutations with KRAS driver mutant contexts sensitize cells to cisplatin.**
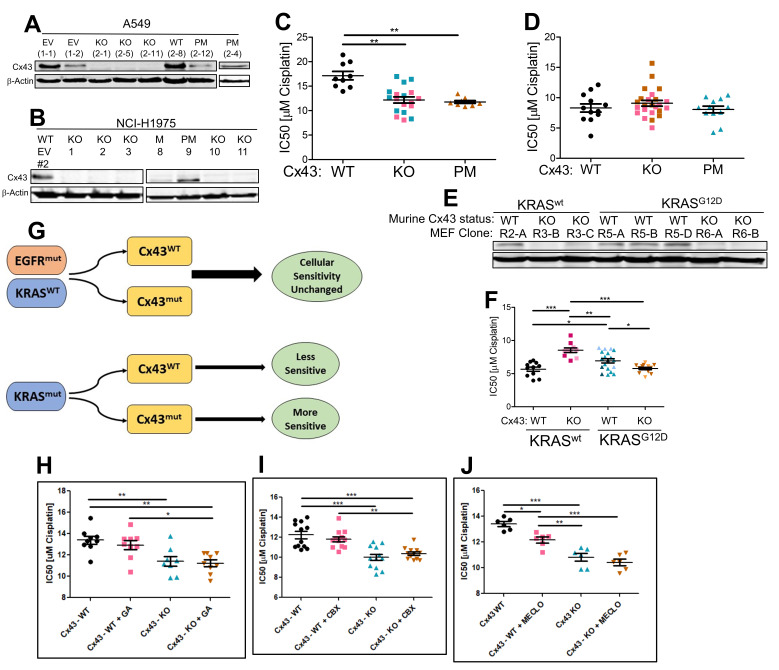
( **A** and **B** ) Immunoblots validating Cx43 expression loss in A549 and NCI-H1975 cell clones post CRISPR editing. ( **C** ) CRISPR-derived A549 cell clones containing Cx43 wild-type (WT), knockout (KO), or point mutant (PM) were treated with cisplatin for 48 hrs. CX43-WT clone: 1-2; Cx43-KO clones: 2-1 (pink), 2-11 (blue); Cx43-PM clone: 2-4. ( **D** ) CRISPR-derived NCI-H1975 cell clones containing edited Cx43 alleles were treated with cisplatin for 48 hrs. Cx43-WT clone: EV#2; Cx43-KO clones: #3 (pink) and #11 (orange); Cx43-PM clones: #9. ( **E** ) Immunoblots validated Cx43 expression loss in the MEF cell clones following Cx43-targeted CRISPR editing. The MEF cells contained either KRAS ^wt^ or KRAS ^G12D^ human transgene in an endogenous murine kras/nras/hras-null background. ( **F** ) CRISPR-derived mouse embryonic fibroblast (MEF) clones containing edited Cx3 alleles were treated with cisplatin for 48hrs. KRAS ^WT^ /Cx43-WT: R2-A; KRAS ^WT^ /Cx43-KO: R3-B (maroon); KRAS ^WT^ /Cx43-KO: R3-C (pale pink); KRAS ^G12D^ /Cx43-WT: R5-A (turquoise); KRAS ^G12D^ /Cx43-WT: R5-B (pale blue); KRAS ^G12D^ /Cx43-WT: R5-D (dark blue/green); KRAS ^G12D^ /Cx43-KO: R6-A (dark orange); KRAS ^G12D^ /Cx43-KO: R6-B (pale orange). ( **G** ) Graphical abstract of the main observation. ( **H** - **J** ) Attempts to phenocopy the chemosensitization effect of Cx43-WT A549 cells using chemical inhibitors of GJIC. 18β-glycyrrhetinic acid (GA) (0.5 μM); Carbenoxolone (CBX) (25 μM); Meclofenamate (MECLO) (25 μM). All IC50 values were calculated and expressed as mean ± SEM. Significance was determined using one-way ANOVA with Bonferroni post hoc tests and expressed as *, p ≤ 0.05; **, p ≤ 0.01; ***, p ≤ 0.001.

## Description

GJIC plays an important role in regulating cell growth and proliferation. Lung cancer cells often have dysregulated GJIC which can have a variety of cellular impacts (Zefferino et al., 2019). Gap junctions are comprised of Cx hexamers embedded in the plasma membrane and Cx43 is one of the Cxs better characterized (Lampe & Laird, 2022). The human lung cancer cell line A549 transfected with a small interfering (si) RNA-targeting Cx43 was more resistant to a first-line chemotherapy cisplatin than the parental A549 cells (Yu et al., 2014). However, siRNA knockdown is prone to unintended off-target effects that may introduce confounding variables (Fedorov et al., 2006; Lin et al., 2019). Additionally, mutations in the oncogenic driver genes like KRAS and epidermal growth factor receptor (EGFR) have been shown to alter cellular sensitivity to cisplatin (Luo & Lam, 2013). It was not yet well understood how these driver mutations would impact Cx43’s chemomodulatory role.

To better investigate how Cx43 may modulate cisplatin sensitivity, we used the CRISPR technology to target the Y17 position of the Cx43 alleles of two lung cancer cell lines that naturally harbor either a KRAS ^G12S^ (A549), or an EGFR ^L858R/T790M^ mutation (NCI-H1975), in view of the fact that KRAS and EGFR mutations are present in 50% of lung adenocarcinomas and are mutually exclusive (Unni et al., 2015) ( **Fig. 1A** and **1B** ). Additionally, we also edited the Cx43 alleles in the RAS-less mouse embryonic fibroblast (MEF) cells (Drosten et al., 2010) transfected with either a KRAS ^WT^ or KRAS ^G12D^ human transgene ( **Fig. 1E** ).

Although previous studies indicated that siRNA-reduced Cx43 expression increases cellular sensitivity to cisplatin, (Yu et al., 2014), we found that the CRISPR-directed loss of Cx43 expression sensitized A549 cells to cisplatin ( **Fig. 1C** ). However, the loss of Cx43 expression did not impact the sensitivity of NCI-H1975 cells to cisplatin ( **Fig. 1D** ). Thus, it appears that the ability of Cx43 to modulate cisplatin sensitivity may be dependent on an underlying KRAS mutation.

We extended our study to include a pair of Ras-less MEF cells stably transfected with either a KRAS ^WT^ or a KRAS ^G12D^ human transgene in the background of lacking endogenous expression of H-, N-, and K-ras murine genes. This model system provides the isogenic tools to validate the requirement of a KRAS mutation for Cx43-dependent alterations of cellular sensitivity to cisplatin. We used CRISPR to inactivate the Cx43 alleles of these MEF cells ( **Fig. 1E** ) and determined that Cx43-KO/KRAS ^G12D^ MEFs were significantly more sensitive to cisplatin than Cx43-KO/KRAS ^WT^ MEFs ( **Fig. 1F** ). It was also observed that Cx43-KO/KRAS ^G12D^ cells were significantly more sensitive to cisplatin than Cx43-WT/KRAS ^G12D^ cells. These observations substantiate our initial findings in the human A549 cell line. Intriguingly, we observed that Cx43-KO in KRAS ^WT^ MEFs appeared to significantly increase cellular resistance to cisplatin.

Cx43 is known to have GJIC-independent activities. We reasoned that if chemical GJIC inhibitors could alone induce the chemosensitization effect on Cx43-WT cells, it would suggest that the chemosensitization effect induced by loss of Cx43 expression is likely dependent on comprised GJIC. To this end, we treated Cx43-WT A549 cells with three known GJIC inhibitors, 18β-glycyrrhetinic acid, carbenoxolone, and meclofenamate ( **Fig. 1H – 1J** ). Only meclofenamate marginally phenocopied the loss of Cx43 expression in sensitizing Cx43-WT A549 cells to cisplatin. While it is tempting to suggest that compromised GJIC may partially mediate the chemosensitization effects induced by Cx43 loss based on the meclofenamate data, more work is needed to solidify this finding.

To conclude, we found that CRISPR-induced loss of Cx43 expression sensitized KRAS mutant cells to cisplatin ( **Fig. 1G** ). However, a chemomodulatory role for Cx43 was not observed in EGFR mutant cells. These findings add new insight to the documented chemomodulatory roles of Cx43 (Murphy et al., 2016; Pridham et al., 2022) and additional studies investigating the mechanistic basis of our observation would be warranted. In view of the relatively small chemomodulatory effect observed in our study, we recognize that a future clinical benefit is unlikely to be obtained by a loss-of-function strategy of Cx43. Nevertheless, the fact that loss of Cx43 expression would exhibit an oncogene-dependent chemosensitization phenotype implicates a complex interaction web between Cx43 and major oncogenes.

## Methods

***CRISPR Mutant Clone Generation***

Human and murine Cx43-Y17-targeting gRNA oligos were obtained from Integrated DNA Technologies (Human -Fw: 5’-CACCGGTTCAAGCCTACTCAACTGC-3’ and Rev: 5’-AAACGCAGTTGAGTAGGCTTGAACC-3’; Murine - Fw: 5’-CACCGGTCCAAGCCTACTCCACGGC-3' and Rev: 5’-AAACGCCGTGGAGTAGGCTTGGACC-3’). gRNA oligomers were annealed, gel-purified (Qiagen, 28704), and ligated into the BsmBI-opened *Lenti-CRISPR-V2 plasmid (Addgene, #52961).* Lentiviruses were produced by cotransection of three plasmids (pVSVg (Addgene 8454), psPAX2 (Addgene 12260), and Lenti-CRISPR-V2) into 293T cells. Two types of Lenti-CRISPR-V2 plasmids were used: gRNA-free (i.e., Empty Vector (EV) control) or the one containing the gRNA targeting Cx43-Y17. Lentivirus-infected cells were selected using 1.5 µg/mL of puromycin (ThermoFisher Scientific, A1113802) for 48-72 hours. Following the puromycin selection of 4 – 5 days, clonal cells for the EV negative control cell clones and Cx43-Y17-targeted clones were isolated by limiting dilution.

***Immunoblotting***

Cell lysates were harvested with 1x Laemmli lysis buffer [0.05 M Tris (ph 6.8), 7.5% Glycerol, 2% SDS, 5% β-mercaptoethanol, 50 mM NaF, 0.5 mM Na _3_ VaO _4_ , 1x P.I. Cocktail (Thermo Scientific, 1862209)] . Protein content was separated using 12% agarose gels and transferred to a nitrocellulose membrane. Antibodies for β-actin (Novus, NBPI-47423) and Cx43 (Cell Signaling Technology, 3512S) were used. Secondary antibodies (Licor 926-32211 and 926-68070) were used to read on a Licor Odyssey scanner.

***Chemosensitivity assays***

Cells (3.5x10 ^3^ ) were plated in each well of a 96-well black-walled plate. Ascending concentrations of cisplatin (Cayman Chemical, 13119) dissolved in cell culture media were added to cells and performed in triplicate. After 48 hours, CellTiter blue reagent (Promega, G8081) was added to each well and fluorescence was read using a Bio-Tek Synergy HT multiplate reader. IC50 calculations based on fluorescence values were determined using GraphPad Prism V5.

***Statistical analysis***

One-way ANOVA and Bonferroni post hoc comparison calculations were used to analyze data for statistical significance as described (Phelps et al., 2019). *, p ≤ 0.05; **, p ≤ 0.01; ***, p ≤ 0.001.

**Photo Documentation**

Pictures were taken with a Licor Odyssey Infrared Imaging System 9120 scanner. Adobe Photoshop 7.0 was used for cropping and careful brightness adjustments. All figures were arranged using Microsoft PowerPoint 2019.

## Reagents

***Cell Culture and Reagents***

A549 and NCI-H1975 cells were obtained from ATCC. The cell lines were cultured in RPMI-1640 (Corning Life Sciences, 10-041-cv) with 10% FBS (VWR 89510-186) and 1x penicillin/streptomycin (100x stock by Corning Life Sciences #30-002-CI). Mouse embryonic fibroblasts (MEF) engineered to exclusively express WT or G12D mutant KRAS transgenes (with no endogenous H-, N-, and K-Ras expression) were obtained from NCI (RPZ25854 and RPZ113). MEF cells were cultured in DMEM (1x, Corning Life Sciences 10-027-cv) in 10% FBS with penicillin (100IU/mL)/streptomycin (100μg/mL). Cells were incubated at 37℃ with 5% CO _2_ .
